# Clinical depression in children and adolescents with sickle cell anaemia: influencing factors in a resource-limited setting

**DOI:** 10.1186/s12887-021-03015-1

**Published:** 2021-12-01

**Authors:** Osita Ezenwosu, Barth Chukwu, Ifeyinwa Ezenwosu, Ndubuisi Uwaezuoke, Christopher Eke, Maria Udorah, Chinedu Idoko, Anthony Ikefuna, Ifeoma Emodi

**Affiliations:** 1 Department of Paediatrics, Faculty of Medical Sciences, College of Medicine, University of Nigeria, Ituku-Ozalla Campus, Enugu, Nigeria; 2 Institute of Maternal and Child Health, University of Nigeria, Ituku-Ozalla Campus, Enugu, Nigeria; 3 Centre for Translational and Implementation Research, University of Nigeria, Enugu, Nigeria; 4 Department of Community Medicine, University of Nigeria Teaching Hospital, Ituku-Ozalla, Enugu, Nigeria; 5 Department of Paediatrics, Annunciation Specialist Hospital, Emene, Enugu, Nigeria

**Keywords:** Factors, Depression, Children, Adolescents, Sickle cell

## Abstract

**Background:**

Sickle cell anaemia (SCA) is the commonest monogenic haematologic disorder resulting from the inheritance of homozygous mutant haemoglobin genes from both parents. Some factors have been identified as important in explaining the variability in depression in sickle cell anaemia (SCA). Information on this is limited in a resource-limited setting like Nigeria. This study aims to determine factors which influence depression in children and adolescents with sickle cell anaemia in a resource-limited setting.

**Methods:**

Systematic random sampling technique was used in this cross-sectional study to select children and adolescents aged 7–17 years at the weekly sickle cell clinic of the University of Nigeria Teaching hospital (UNTH) Enugu, Nigeria. Pretested, structured questionnaire was used to collect sociodemographic and disease severity data while depression was assessed using the Children’s Depression Inventory.

**Results:**

Age and educational level had significant positive linear relationships with depression (r = 0.253, *p* = 0.02; r = 0.225, *p* = 0.04 respectively) while gender (χ2 = 0.531, *p* = 0.466), socioeconomic status (χ2 = 0.451, *p* = 0.798) and disease severity (χ2 = 0.422, *p* = 0.810) had no relationship with depression in children and adolescents with SCA.

**Conclusion:**

Depression in children and adolescents with SCA increased with increasing age and educational level. Psychological evaluation should be integrated into routine assessment of children with SCA during their follow up visits as they get older and progress in class.

## Introduction

Sickle cell anaemia (SCA) is the commonest monogenic haematologic disorder which results from the inheritance of mutant haemoglobin genes from both parents [1, 2]. SCA affects physical, skeletal and sexual developments [3] which in addition to increased disease-related morbidity and severity may lead to psychosocial disturbances [4]. Depression remains the most frequent psychological problem encountered in people with SCA with resultant social withdrawal, poor relationships and poor school performance [4, 5]. Unlike in developed countries where the prevalence of depression in SCA is low [6], the prevalence is high in Nigeria [7] and other resource-limited settings [8, 9] ranging from 22 to 86%.

While depression has been shown to be the commonest of the psychosocial problems experienced by people with SCA [4], some factors may explain the presence of depression in children with SCA. These factors may include:

### Age

Generally, adolescents report more depressive symptoms than younger children because of the peculiar stressors they experience [10] and adolescence is a critical developmental period marked by many physical, emotional, and psychological challenges during which new sense of identity is developed and independence is achieved [11]. This same age effect seen in general population is also observed among adolescents and children with SCA. However, compared to the general population, adolescents with SCA report more depressive symptoms than younger children, suggesting the possibility of unique challenges among them [12]. Probably, this could be due to their experience of disease complications including delayed puberty, short stature, and fatigue which cause additional stress for adolescents with SCA because of their enormous influence on adolescents’ identity development, social role participation and independence [12]. Thus, in addition to the normal stressors that all adolescents face, those with SCA experience additional disease related challenges that can lead to depression during this crucial developmental period.

However, despite the expected higher incidence of depression in adolescents compared to children, studies from different parts of world have documented lack of association between age and prevalence of depression in SCA patients [6, 13].

### Gender

The gender-related patterns of depression in the general population show more depressive symptoms in adolescent girls than their adolescent male counterparts [14]. In SCA, there are diverse findings regarding the effect of gender on depression. While some authors found no relationship between gender and occurrence of depression [6, 13, 15–17], others noted significantly higher depression scores in females [18, 19].

Though controversy surrounds the effect of gender on depression in the studies, the inconsistencies were noted in the studies involving adults as their subjects [15–17, 19]. Studies with children and adolescents as their subjects had consistently noted gender as non-predictive factor of depression [6, 13].

### Educational level

Like other sociodemographic parameters, educational level of the subjects shows variable effects on occurrence of depression in individuals with SCA. Thus, while in some studies educational levels were not significant predictors of depression [16, 17], others identified levels of formal education as predictors of depression symptoms [18, 19] even as SCA patients with lower education showed increased risk of being depressed [15].

In these studies, the patients’ formal educational level was the variable used. However, in children and adolescents who are mostly still in school, it is recommended that their parental formal educational level may be used for their assessment since most parents have achieved their highest level [20].

### Occupation/income level of parents

In the general population, there is an inverse relationship between income and prevalence of depression [21] as family income has proven to be a protective factor against depression for both men and women [22]. Interestingly, in SCA patients, studies showed that family income significantly predicted the likelihood of depression as low income is significantly associated with severe depression [17–19]. However, other studies [15, 16] found that this pattern of relationship was not always consistent. Therefore periodic re-assessment of the relationship between income level and depression is important and has been recommended [21].

In children and adolescents with SCA, it is proper to assess them using their parents or care-giver’s income.

### Socio-economic status of the family

There exists a long-held belief that there is an association between depression and socioeconomic status (SES). Akhtar and Landeen [21] has since shown that significant association existed between occurrence of depression and SES. In a further multivariate analysis by the workers [21], SES showed strong relationship with the occurrence of depression. Published researches indicate that despite differences in definitions and measurements of SES, the likelihood of depression in the lowest SES family group is twice as much as that found in the highest SES group [23, 24].

### Disease severity in SCA

Severity of SCA is assessed using clinical and laboratory parameters as well as cumulative incidence of complications [25].

Unlike the laboratory indices, clinical parameters in SCA directly affect the psychosocial function and most authors employ it in relating disease severity in SCA with depression [17–19]. Using this, Laurence et al. [26] noted that the odds of having elevated depressive symptoms were 1.78 (Cl: 0.94, 3.38) for those with high clinical SCA severity scores compared to those without SCA. Previous studies have found elevated depressive symptoms in SCA patients who report more frequent painful sickle cell episodes [17, 19]. Depression has also been associated with an increased number of hospitalization in the preceding 1 year [17–19]. These variables reflect disease severity which is noted to elicit depressive symptoms [19].

Experts had predicted that by 2030, depression alone is likely to be the third leading cause of disease burden in low-income countries and the second highest cause of disease burden in middle-income countries [27]. Evidence still shows that depression and other mental health disorders are highly neglected in low-resource settings [28]. Researches on depression and factors that could lead to depression in such settings may help in early identification of such factors with possible roles in prevention and reduction in prevalence of depression. To the best of our knowledge, the factors influencing depression among children with SCA has not been determined in a resource-limited setting like Nigeria. This study, therefore, aims at determining the factors influencing the presence of depression in children and adolescents with SCA in Enugu, South-east Nigeria.

## Materials and methods

The study population for this cross-sectional, analytical study were children and adolescents with SCA attending the weekly sickle cell clinic of the University of Nigeria Teaching Hospital (UNTH), Enugu, Nigeria and aged 7–17 years. The UNTH serves as a referral center for all the States in the southeastern geo-political zone of Nigeria. Using systematic random sampling method and sample size determination formular for comparing two proportions [29], 84 children and adolescents with SCA who satisfied the inclusion criteria (have been attending sickle cell clinic for at least 6 months, without other chronic diseases, gave assent while parents gave consent) were recruited for the study. The number was derived when Zα = standard deviate at α probability (1.96), Zβ = standard deviate at β probability (0.84), P_1_ = proportion of SCA children with depression in our environment = 0.54 [30], P_2_ = estimated proportion expected to have depression assuming a 22% difference [31] and 10% attrition. The controls were 84 age and sex matched peers recruited at the outpatient clinic during their follow up following recovery from acute illnesses. These controls tested AA as genotype with Hb electrophoresis and without other chronic conditions.

Depression was assessed with the children’s depression inventory (CDI) adapted from Kovacs [32]. The CDI is self-report instrument used to screen for depression in children and adolescents aged 7–17 years. It consists of 27 items which yield scores for five subscales namely- negative mood, interpersonal problems, ineffectiveness, negative self-esteem and anhedonia. Each item has 3 response options from which the child selects the one that most closely reflects his or her thoughts and feelings over the previous 2 weeks. The options are scored 0 to 2 points with a highest possible total score of 54. A score of ≥19 reflects clinical depression [33].

An interviewer administered questionnaire was used to collect socio-demographic data (age, sex, place of residence, class in school, highest educational level of parents, and occupation of parents) from the subjects and controls. Their socioeconomic status were determined using the occupation and highest educational attainment of both parents proposed by Oyedeji as described by Ikefuna and Emodi [20]. Class I represented the highest social class while class V represented the lowest and these were classified into upper (I & II), middle (III) and lower (IV & V) socioeconomic status [20].

Clinical disease severity was evaluated using the pre-tested and validated, structured interviewer administered questionnaire adapted from Adegoke and Kuti [34]. A total of thirteen parameters were evaluated. Items in the previous 12 months (number of painful episodes, transfusions and hospitalizations) were scored according to their frequency of occurrence (0 = None, 1 = once, 2 = twice or thrice, 3 = > thrice); Items in present state (splenic enlargement and liver enlargement) were scored by severity (0 = < 5 cm, 1 = 5-10 cm, 2 = > 10 cm) while items in lifetime complications (complications such as cerebrovascular disease, acute chest syndrome, avascular necrosis, Pneumococcal meningitis, osteomyelitis, gall stone, chronic leg ulcer and priapism) were scored based on their absence or presence (0 = absent, 1 = present). The total calculable score was 0–21 and the disease severity was classified as mild when the total score was ≤6, moderate with score of 7 to 12 while total score > 12 represented severe disease.

Means were compared using Student’s t-test, while the Chi-squared test was used to compare proportions. The relationship between numerical variables were tested with the Pearson’s correlation coefficient whereas Chi square was used to test for association between categorical variables. The level of significance was taken as p <0.05.

The Health Research Ethics Committee of UNTH, Enugu gave ethical approval for this study while informed assent and consent were obtained respectively from the participants and their caregivers or the legally accepted representative before enrollment.

## Results

Eighty four children and adolescents with SCA who met the eligibility criteria were recruited as subjects for the study while their control group were 84 age and sex matched peers. Table 1 shows the sociodemographic characteristics of the participants. The proportion of children in different age groups and genders were similar in subjects and controls. Also the proportions in the educational categories were similar (χ^2^ = 2.19, *p* = 0.534). Most of the participants (45.2% of the subjects and 47.6% of the controls) belong to the middle socioeconomic class and this difference was not significant (χ^2^ = 1.061, *p* = 0.588).

**Table 1:** Sociodemographic characteristics of the study population

Variables	Subjects	Controls			
	No. (%)	No. (%)	χ^2^	df	*p*
**Age groups**					
Children (7–12 years)	47 (56.0)	47 (56.0)	0.000	1	1.000
Adolescents (13–17 years)	37 (44.0)	37 (44.0)			
**Gender**					
Males	47 (56.0)	47 (56.0)	0.000	1	1.000
Females	37 (44.0)	37 (44.0)			
**Educational level**					
Early Primary (Primary 1–3)	18 (21.4)	20 (23.8)	2.190	3	0.534
Late Primary (Primary 4–6)	24 (28.6)	20 (23.8)			
Junior Secondary	24 (28.6)	19 (22.6)			
Senior Secondary	18 (21.4)	25 (29.8)			
**Socio-economic Status**					
Upper	29 (34.5)	32 (38.1)	1.061	2	0.588
Middle	38 (45.2)	40 (47.6)			
Lower	17 (20.2)	12 (14.3)			

The mean depression scores was 7.54 ± 5.97 for the subjects and 6.77 ± 4.85 for the controls but the difference was not statistically significant (χ^2^ = 0.908, df = 66, *p* = 0.365). Seven (8.3%) subjects and 2 (2.4%) controls had clinical depression but their difference in proportion did not reach statistical significance (Fisher’s χ^2^ = 2.935, *p* = 0.168).

Effect of sociodemographic and disease severity factors on depression among children and adolescents with SCA are shown below.

**Fig. 1:**
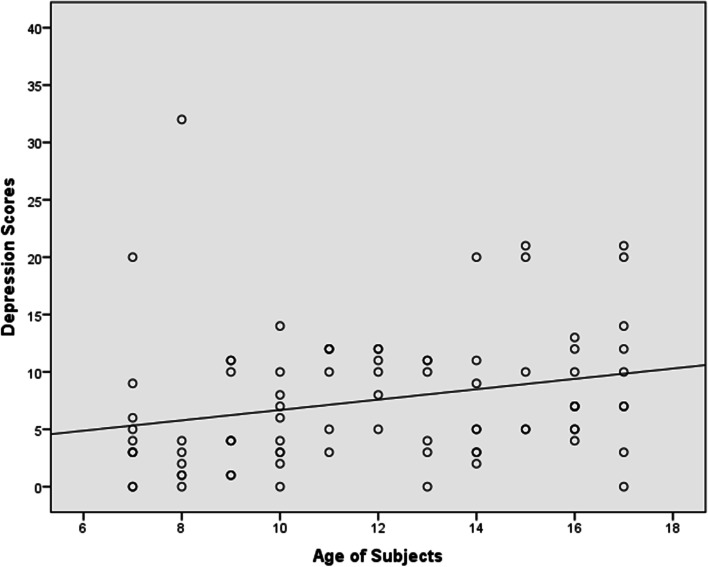
Relationship between depression scores and age of subjects

Figure 1 is a scatter plot with line of best fit of the relationship between depression scores and age of subjects. The depression scores and age had a statistically significant positive linear relationship (r = 0.253, *p* = 0.02).

The distribution of depression in relation to sex of the subjects is shown in Table 2. Three (6.4%) of the 47 males compared with 4 (10.8%) of the 37 females had depression diagnostic scores while 44 and 33 respectively had normal scores. However, there was no statistically significant association between sex and level of depression (χ^2^ = 0.531, *p* = 0.466).

**Table 2:** Distribution of subjects with depression according to gender

Gender	Depression				
	No	Yes	χ^2^	df	*p*
Males (*n* = 47)	44 (93.6)	3 (6.4)			
			0.531	1	0.694*
Females (*n* = 37)	33 (89.2)	4 (10.8)			

*Fisher’s test

**Fig. 2:**
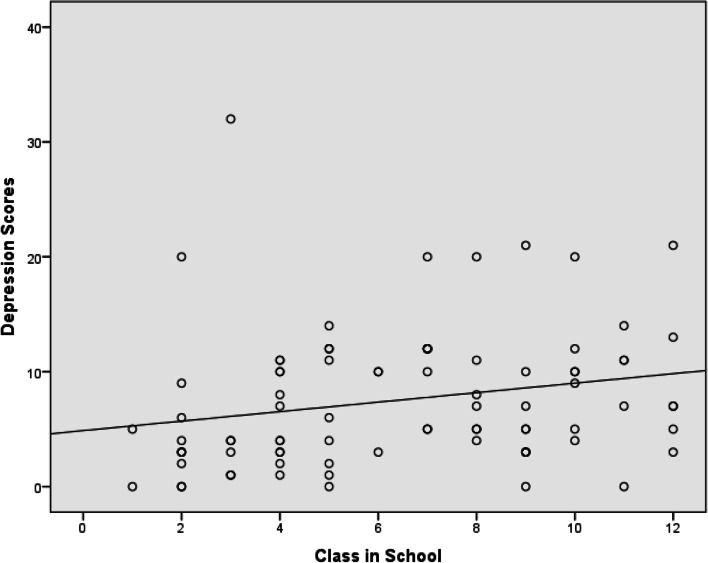
Relationship between depression scores and educational level of subjects. Legend: 1 = Primary 1; 2 = Primary 2; 3 = Primary 3; 4 = Primary 4; 5 = Primary 5; 6 = Primary 6; 7 = Junior Secondary 1; 8 = Junior Secondary 2; 9 = Junior Secondary 3; 10 = Senior Secondary 1; 11 = Senior Secondary 2; and 12 = Senior Secondary 3

The scatter plot with line of best fit of the relationship between depression scores and educational levels of the subjects showed a progressive increase in depression scores with increasing educational level (Fig. 2). The 2 variables showed a statistically significant positive linear relationship (r = 0.225, *p* = 0.04).

**Fig. 3:**
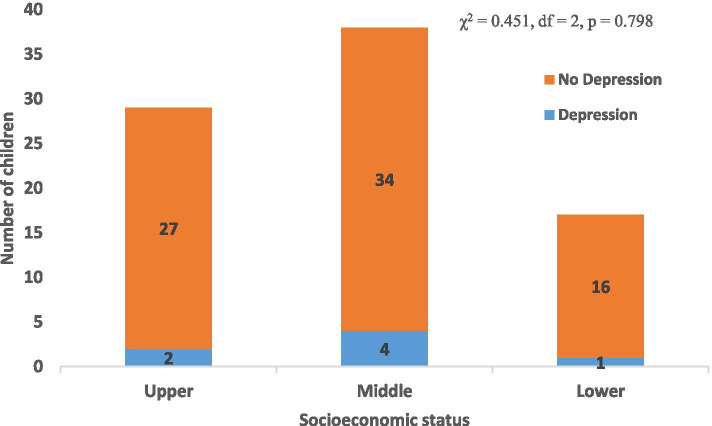
Association between depression and socio-economic status of the subjects

Figure 3 shows the association between depression and socioeconomic status of the subjects. Two (6.9%) of 29 subjects in upper class, 4 (10.5%) of 38 in middle class and one (5.9%) of 17 in lower class had depression. However, the overall relationship between depression and family socioeconomic class was not statistically significant (χ^2^ = 0.451, *p* = 0.798).

**Fig. 4:**
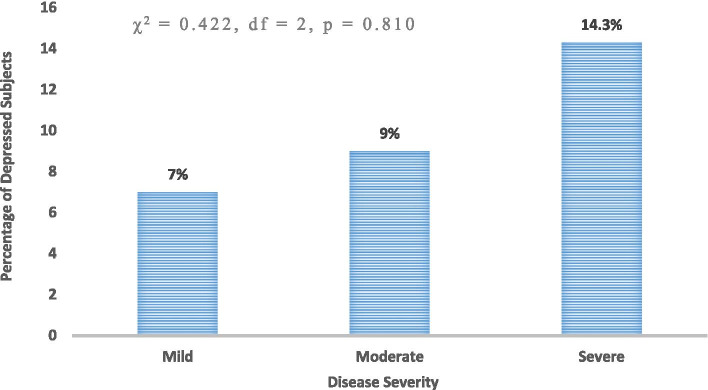
Association between depression and disease severity among the Subjects

The association between depression and disease severity among the Subjects is shown in Fig. 4. Seven percent (4/55) of those with mild disease had depression, whereas 9% (2/22) of those with moderate disease had depression while 14.3% (1/7) subject(s) with severe disease was depressed. Though the proportion with depression increased with increasing disease severity, there was no statistically significant association between disease severity and depression (χ^2^ = 0.422, *p* = 0.810).

## Discussion

This study aimed to determine factors which influence depression in children and adolescents with SCA in a low-resource setting. Among the determined factors, age and educational level had positive relationships while gender, socioeconomic status and disease severity had no relationship with depression in children and adolescents with SCA.

The absence of an association between gender and depression among children and adolescents in this study interestingly added to the inconsistency noted in previous researches. Thus while Friedberg and Sinderman [35] argued that females are expected to score higher on CDI and Hasan et al. [19] believed that female sex is an important predictor of depression in SCA, other workers [6, 13, 15, 17, 36] documented lack of association between sex and depression in SCA. The controversy surrounding the effect of gender on depression may be due to differences in the age groups of recruited SCA patients as adolescent girls are noted to have higher rates of depression than boys while pre-adolescence attenuate the gender differences [35]. Thus, similar to our study, studies with children and adolescents as their subjects consistently found gender as non-predictive of depression [6, 13].

Furthermore, recruitment of children and adolescents may allow the effect of age to be determined compared to recruitment of homogenous groups like young adults or adults. Researchers had reported that older children endorsed more symptoms on the CDI and total CDI scores significantly correlated with children’s age [35]. Our study found a significant positive correlation between age and CDI scores. The age range in this study was 7–17 years while previous studies on effect of age recruited much older children and documented lack of relationship between age and depression in individuals with SCA [15, 16, 18]. This finding can be explained by the fact that aside more depressive symptoms in adolescents because of the peculiar stressors they face from physical, emotional, and psychological challenges [10, 11], those with SCA experience additional disease related challenges such as delayed puberty, short stature, fatigue and leg ulcers which can lead to depression [12].

Interestingly, there was a significant positive linear relationship between educational level and depression scores of the SCA children and adolescents. This is expected since their educational level naturally increase with increasing age. This is inconsistent with the observation of Hasan et al. [19] who noted that less than high school education significantly predicted the likelihood of depression compared to above high school. Alhamoud et al. [15] and Altaitoon et al. [18] also found a significant association between lower education and depression. The difference between our finding and that of previous studies may be paradoxical considering that the lower age category in these studies (adolescents) corresponds with the higher age category in our study. Hence since educational level is usually affected by age, the adolescents will likely be in lower educational level in the previous studies while in higher educational level in this study but still report their typical higher depressive symptoms in both settings. Thus, as has been suggested, psychological evaluation may be incorporated into the protocol for management of children with SCA as they progress academically [37].

The results demonstrated lack of association between depression and socio-economic status of the subjects. This finding disagrees with long held belief that there is an association between depression and socioeconomic status [21]. It also contrasted with previous observations that depression was generally more common among people in low socioeconomic group [24]. Unlike other parents belonging to low socioeconomic classes, parents of children with SCA may be providing them with extra care which may allay depressive tendencies. Hence, Sehlo and Kamfar [13] found that higher level of parental support is a significant predictor of low depression state in children with SCA.

It is noteworthy that despite the perceived effect of SCA disease severity on psychosocial function [26], and the proportion of SCA people with depression increasing with increasing disease severity in this study, there was lack of association between depression and disease severity. This finding is in keeping with the observation by previous researchers that, using CDI, disease severity is not a predictor of depression [13]. In contrast, Hasan and colleagues [19] assessed measures of severity and found each a significant predictors of depression. The lack of relationship in this study may not be unrelated to the recruitment done in the clinic when they are stable and may not fully recount previous experiences. Again, the result may have been affected by the period considered in CDI tool used for assessment of depression. As feelings and thoughts in the preceding 2 weeks are reported, some of the children with severe disease may not fully recall them while others may feel restricted from expressing the thoughts and feelings beyond preceding 2 weeks.

A limitation of this study is the use of a single setting. Although UNTH serves a large geographic region, which encompasses rural and urban centers, statements about generalizability may be conservative. A multi-site study would have improved generalization of the study.

## Conclusion

In this study, depression scores in children and adolescents with SCA increased with their increasing age and educational status. Thus, as children with SCA get older and progress in class, they need clinical psychological complement to their routine care. Therefore, in low-resource settings where there are limited availability of psychiatrists, there may be need to train the primary care physicians and Paediatric Haematologists to provide such service. Also, Counsellors and child’s Psychologists in other settings may find the outcome of this study useful as it could equip them with the information that could be communicated to the caregivers and parents of children with SCA.

## Data Availability

The datasets generated and/or analyzed during the current study are not publicly available due to confidentiality policies but are available from the corresponding author on reasonable request.

## Abbreviations

SCA: Sickle Cell Anaemia
SES: Socio-Economic Class
UNTH: University of Nigeria Teaching Hospital
CDI: Children’s Depression Inventory
CVD: Cerebro-Vascular Disease
ACS: Acute Chest Syndrome
AVN: Avascular Necrosis
